# Risk Factors Associated with Cutaneous Reactions Following COVID-19 Vaccine Immunisation: A Registry-Based Case-Control Study

**DOI:** 10.21315/mjms2024.31.3.10

**Published:** 2024-06-27

**Authors:** Hwei Lin Teh, Thamron Keowmani, Min Moon Tang

**Affiliations:** 1 Pharmacy Department, Hospital Kuala Lumpur, Kuala Lumpur, Malaysia; 2 Pharmacy Department, Hospital Wanita dan Kanak-Kanak, Sabah, Malaysia; 3 Department of Dermatology, Sarawak General Hospital, Sarawak, Malaysia

**Keywords:** pandemic, COVID-19 vaccine, adverse effects, risk factor, cutaneous reaction, hypersensitivity

## Abstract

**Background:**

In Malaysia, following extensive COVID-19 vaccination, Hospital Kuala Lumpur reported an increase in cutaneous reactions post-immunisation. To understand this, a case-control study was initiated to identify potential risk factors.

**Methods:**

This registry-based, unmatched case-control study encompasses all adverse event following immunisation (AEFI) reports associated with COVID-19 vaccines, received by the Department of Pharmacy at Hospital Kuala Lumpur through the Malaysian Adverse Drug Reactions Advisory Committee (MADRAC) AEFI reporting forms. Twenty-four potential risk factors were evaluated, including demographic information, medical history, food allergies, COVID-19 vaccination history and prior SARS-CoV-2 infection, were evaluated using MADRAC AEFI reporting forms. Odds ratio (OR) with 95% confidence interval (CI) were estimated using univariable and multivariable logistic regression.

**Results:**

Cutaneous reactions were more frequent in middle-aged females, especially after the first COVID-19 vaccine dose. These reactions, primarily mild and generalised, included pruritus and urticaria. Notably, 52% were delayed reactions (more than 4 h post-vaccination). Factors associated with increased risk of cutaneous reaction following COVID-19 immunisation included history of seafood and shellfish allergy (adjusted odds ratio [adjOR]: 2.11; 95% CI: 1.12, 3.96; *P* = 0.020), history of vaccine allergy (adjOR: 4.07; 95% CI: 1.44, 11.54; *P* = 0.008), past dermatological diseases (adjOR: 5.48; 95% CI: 2.03, 14.78; *P* = 0.001), and past medication allergy (adjOR: 2.12; 95% CI: 1.36, 3.31; *P* = 0.001).

**Conclusion:**

Self-reported histories of allergies to vaccines, foods or medications were found to increase the likelihood of cutaneous reactions following COVID-19 vaccination. These reactions, which were predominantly mild, did not hinder the administration of the second vaccine dose. The majority of reactions occurred after the first dose, manifesting as generalised pruritus and urticaria. They were effectively managed with oral antihistamines and low-dose corticosteroids, thereby avoiding the need for hospitalisation.

## Introduction

In December 2019, a novel coronavirus disease (COVID-19), caused by severe acute respiratory syndrome coronavirus 2 (SARS-CoV-2), emerged and spread globally, leading the World Health Organization (WHO) to declare it a pandemic in March 2020. Malaysia began its mass immunisation campaign against SARS-CoV-2 on 24 February 2021, as part of the National Immunisation Programme for COVID-19. Vaccination continues to be one of modern medicine’s most effective public health interventions (PICK). Since then, five COVID-19 vaccines, namely Comirnaty^®^ (Pfizer), CoronaVac (Sinovac), Vaxzevria (AstraZeneca), Convidecia (Cansino) and Covilo (Sinopharm) have been used in Malaysia. All these vaccines meet the high standards for quality, safety and efficacy set by the National Regulatory Agency (NPRA). All these vaccines meet the high standards for quality, safety and efficacy set by the NPRA.

Following this mass vaccination programme, the NPRA received a surge in reports of adverse events following immunisation (AEFI). The total of AEFI reports received was 26,613 as of September 2022, which is equivalent to 370 reports per 1,000,000 doses administered. The vast majority (93%) of the reported AEFIs in Malaysia were non-serious. One of the commonly reported adverse events was cutaneous adverse reactions (1). These cutaneous reactions were reported as broad spectrum and could be immediate or delayed (2–5). In fact, it is not uncommon, as clinical studies and post-marketing phases have reported adverse reactions affecting the skin and mucous membranes in 0.63%–1.6% of COVID-19 vaccine recipients (6–8). However, the rate of self-reported acute allergic reactions is slightly higher, at up to 2.10%, with messenger RNA (mRNA)-based COVID-19 vaccines (9).

Given the importance of widespread vaccination programmes in combating this public health crisis, understanding the adverse skin and allergic reactions to the approved vaccines is crucial. A history of allergies is common in individuals with confirmed anaphylaxis to mRNA-based COVID-19 vaccines. Previous studies have shown that a history of vaccine allergy, medication or other allergens is associated with self-reported allergic reactions within the first 3 days after receiving either dose 1 or dose 2 of mRNA-based COVID-19 vaccine (10). However, the risk factors for cutaneous reactions following COVID-19 vaccine immunisation are not yet well established, although a few studies have attempted to identify host and vaccine constituent risk factors associated with post-vaccination allergic reactions in other vaccines (11–15). This study aims to describe the characteristics of cutaneous reactions following COVID-19 vaccine administration and to investigate potential risk factors.

## Methods

### Study Design and Setting

This registry-based, unmatched case-control study encompasses all AEFI reports received by the Department of Pharmacy at Hospital Kuala Lumpur via Malaysian Adverse Drug Reactions Advisory Committee (MADRAC) AEFI reporting forms. The National Pharmaceutical Regulatory Agency (NPRA) mandates such reporting of AEFIs as part of passive vaccine surveillance. These forms facilitate the collection of data on AEFI types, demographics, onset, AEFI severity, comorbidities and related laboratory values from patient medical records. The study included all adults aged 18 and above who presented to Hospital Kuala Lumpur with adverse events within 90 days following COVID-19 immunisation, from 1 April 2021 to 30 April 2022.

### The Selection of Cases and Control

The geographic area for this study was chosen because the majority of adverse events following immunisation were expected to present at Hospital Kuala Lumpur, a 2,300-bed tertiary hospital. Cases were primarily identified by physicians in the Department of Accident and Emergency, but they could also be identified by physicians assigned at the vaccination centre, after a patient was admitted to the inpatient wards or during outpatient clinic visit. Any MADRAC reports with cutaneous adverse reactions reported within 90 days post-COVID-19 immunisations were included. The unmatched controls were the remaining MADRAC reports without cutaneous adverse reactions. We excluded MADRAC reports involving cases that were brought in dead.

### Measurement of Study Variables

Patient demographics, adverse event descriptions, the presence and absence of cutaneous manifestations, comorbidities, histories of vaccine or medicine allergies, food allergies and other diseases were captured using the MADRAC AEFI reporting forms.

### Statistical Analysis

All analyses were carried out using SPSS version 24.0. Demographic data were analysed using descriptive analysis, where categorical variables were expressed as frequencies and percentages, while continuous data were reported as means (standard deviation) for normally distributed data or medians (interquartile range) for non-normally distributed data.

To explore the risk factors associated with cutaneous reactions, potential predictive variables including demographic information, comorbidities, types of vaccines and histories of allergens were investigated using simple logistic regression analysis. The reference level was patients having no cutaneous reaction. Potential variables with a *P*-value < 0.25 were selected for inclusion in multiple logistic regression. The methods used for the selection of variables to be included in the model were forced entry, forward stepwise and backward stepwise. In this process, the probabilities of entry (Pe) and removal (Pr) were predetermined as 0.05 and 0.1, respectively, throughout the entire variable selection process. The preliminary final model was checked for multicollinearity and interaction terms, while the final model was checked for model fitness. Any statistically significant adjusted odds ratio (adjOR) reflecting associations not previously reported in the literature were adjusted for multiple testing using the Bonferroni method (16). The results were presented as regression coefficients (b) with standard error (SE), odds ratio (OR), adjOR with their 95% confidence interval (CI) and corresponding *P*-values. Statistically significant results were defined as those where the *P*-value was < 0.05.

The sample size was calculated using G-Power software. The power (1–β) was set at 0.8 to have an 80% chance of detecting a difference if it truly exists. The level of significance, α, was determined at 0.05. All statistical analyses were two-sided tests. The OR was based on a study by Rouleau et al. (10, 17), where food allergy had an adjOR of 3.84 for anaphylaxis reaction secondary to monovalent AS03-adjuvanted pandemic influenza vaccine. Studies from influenza vaccines were used instead of COVID-19 vaccines as data for risk factors are still limited. A total of 176 samples were required from the case group.

## Results

### Demographics, Characteristics, Clinical Presentation and Dermatological Findings of Cases

There were a total of 814 subjects in this study: 602 controls and 212 cases. The demographic distribution, characteristics, clinical presentation and dermatological findings of the cases are shown in Table 1. Most patients (80.2%) with cutaneous reactions were middle-aged, with a median age of 37 years old and predominantly female (71.2%). Of these, 195 patients (92%) received the same type of COVID-19 vaccines (i.e. homologous vaccination) and only 3 (0.1%) had a history of SARS-CoV-2 infection. Regarding a history of allergies, 46 (21.7%) reported a history of allergy to medication, 11 (5.2%) had a history of allergy to vaccines and 27 (12.7%) reported a history of food allergy. The reported cutaneous reactions were predominantly mild (79.7%) and generalised skin reactions (66.9%), with pruritus and urticaria being the most common manifestations, 52% of the cutaneous reactions were delayed (> 4 h).

### Univariable and Multivariable Logistic Regression

The univariable analysis included 24 potential risk factors, of which 13 met the statistical threshold (*P* < 0.25) for inclusion in the multivariable analysis (Table 2). The variables significantly associated with cutaneous reactions, as outlined in Table 2, included female gender, repeated doses of COVID-19 vaccination, history of any food allergy, history of seafood or shellfish allergy, history of medication allergy, history of vaccine allergy and history of dermatological skin disease. After the multivariable logistic regression, 4 of the 13 potential risk factors were retained in various models. The factors retained in the final model are shown in Table 3, and it showed that patients with a history of vaccine allergy had increased odds of having a cutaneous reaction after COVID-19 immunisation by 4.07 times compared to patients without such history (adjOR: 4.07; 95% CI: 1.44, 11.54; *P* = 0.008), while patients with a history of seafood and shellfish allergy had 2.11 times the odds of cutaneous reactions (adjOR: 2.11; 95% CI: 1.12, 3.96; *P* = 0.020). Similarly, patients with past dermatological diseases and past medication allergies had 5.48 times (adjOR: 5.48; 95% CI: 2.03, 14.78; *P* = 0.001) and 2.12 times (adjOR: 2.12; 95% CI: 1.36, 3.31; *P* = 0.001) higher odds of cutaneous reactions, respectively.

## Discussion

Our study has provided significant insights into the nature and risk factors associated with cutaneous reactions following COVID-19 vaccination. The data demonstrate that the majority of these skin reactions were mild in severity (79.7%) and primarily characterised as generalised skin reactions (66.9%), with pruritus and urticaria being the most prevalent manifestations. Interestingly, over half of the cases (52%) experienced delayed cutaneous reactions, occurring more than 4 h post-vaccination. Examination into potential risk factors revealed a statistically significant association between cutaneous reactions post-COVID-19 vaccination and a history of specific allergies such as seafood and shellfish, past vaccine allergies and past dermatological diseases.

The majority of patients experiencing cutaneous reactions after COVID-19 vaccination were middle-aged, especially after the first dose of the vaccine, similar to findings in two other studies by Farinazzo et al. (18) and Grieco et al. (19), although one study reported a higher rate of cutaneous reaction following the second dose (20). Fifty-two percent of cutaneous reactions were delayed (> 4 h), akin to findings from a previous study based on a registry from Massachusetts General Hospital (20), where most cutaneous adverse reactions were delayed cutaneous reactions.

The cutaneous reactions were predominantly mild (77.8%) to moderate (13.2%), aligning with findings from a systematic review of 17 studies on cutaneous adverse reactions (21). They were also generally not serious (86.3%), with only one case resulting in permanent disability, a case of zoster flare-up leading to post-herpetic neuralgia, which has been reported as rare (18, 20, 22). A systematic review also documented that cutaneous side effects of COVID-19 vaccines appear to be more common in women, similar to our study’s findings where up to 71.2% of cutaneous adverse events occurred in females. This difference may be multifactorial. Studies have shown that gender differences can affect immune response, with women exhibiting elevated humoral and cell-mediated responses to vaccines, potentially leading to a higher rate of cutaneous reactions (23, 24). Reporting bias may also play a role, as women constitute the majority of the healthcare workforce at Hospital Kuala Lumpur, who were also the first target group to be vaccinated against COVID-19 (20, 25). Additionally, women are generally more aware of health situations and skin conditions (26).

The top 3 presentations of cutaneous skin reactions were pruritus, urticarial rash and maculopapular rash, though rare skin reactions such as zoster and purpura were also observed. Interestingly, generalised cutaneous reactions were more common (67%) in our study, contrasting the findings from a systematic review by McMahon et al. (20) and another article review by Sun et al. (25), where the majority were local injection site reactions. This discrepancy may be due to reporting bias, as local reactions are considered common and healthcare physicians tend not to report them.

The majority of cutaneous reactions occurred in mRNA-based vaccines (59.8%), with the higher number of cases expected because approximately 62% of our COVID-19 vaccines administered to the Malaysian population were Comirnaty^®^. Of the 112 patients who experienced cutaneous reactions during their first dose of COVID-19 vaccine, 84 patients (75%) proceeded to take their second dose. Of the 71 patients who experienced a cutaneous reaction to the second dose, only 40 patients (56%) proceeded to take the booster dose. Reported cutaneous reactions did not prevent most patients from taking the second dose, but they did prevent some from getting the booster vaccines. The proportion of patients proceeding to take subsequent doses after a cutaneous reaction was much lower than what other studies reported, where more than 90% of patients went ahead with subsequent doses despite cutaneous reactions (10, 20). Lack of eligibility for subsequent doses after an allergy screening, scheduling issues or individual vaccine hesitancies may be some reasons behind incomplete immunisation in the Malaysian context. Recent research showed that the second dose of an mRNA-based COVID-19 vaccine could be safely given even to people who experienced immediate and possibly allergic reactions following the first dose (27). Future audits are necessary to fully understand the causes of delays or failures in completing booster doses of COVID-19 immunisation.

Despite extensive exploration of risk factors, only four variables were found to be associated with cutaneous reactions: history of vaccine, seafood and shellfish allergy. This association was demonstrated by Li et al. (10), where a cohort study found that self-reported history of high-risk allergy to mRNA-based COVID-19 was associated with an increased risk of allergic reactions within 3 days of mRNA-based COVID-19 vaccination (adjOR: 2.46; 95% CI: 1.92, 3.16) and this was also demonstrated in few other studies (28–30). Self-reported history of high-risk allergy in this study was defined as a previous severe allergic reaction to a vaccine, an injectable medication or other allergen. This association may be due to a heightened anticipation or appreciation of possible allergic symptoms by the patient or vaccine provider.

This study also found that patients with a history of vaccine allergy were four times more likely to experience cutaneous reactions following COVID-19 vaccination. A recent study of 429 highly allergic individuals who received the Pfizer-BioNTech vaccine under medical observation identified a much higher rate of minor allergic reactions (31), which may be attributed to excipients rather than vaccine active ingredients (6, 32). Although no study has demonstrated an association between seafood or shellfish allergy and adverse cutaneous or allergic reactions following COVID-19 vaccination, our study found that patients with seafood or shellfish allergies had twice the odds (adjOR: 2.11; 95% CI: 1.12, 3.96; *P* = 0.020) of experiencing a cutaneous reaction. A review by Selvaraj et al. (33) mentioned that allergies to egg, fish, milk, shellfish and tree nuts are due to water-soluble glycoproteins, which are also present in nucleoside-modified mRNA vaccines (BNT162b2 and mRNA-1273) when translated into fragments of the spike glycoprotein of SARS-CoV-2, potentially triggering an anaphylaxis reaction in some vaccinated individuals.

Polyethylene glycol (PEG), which is an ingredient in the mRNA-based vaccines, has infrequently been linked to allergic reactions to other PEG-containing goods and drugs. Polysorbate, a component of the viral vector vaccine that shares structural similarities with PEG, has also been sporadically linked to anaphylactic reactions to medications containing polysorbate. Both of which are recognised as major allergenic or immunogenic excipients (34–36). Given that PEG and polysorbates are common in various medical products, including creams, ointments, lotions, and tablet medications, an association between cutaneous reactions in patients with a history of medication allergies is possible, although PEG allergy incidence is very rare (37). In fact, dose-response relationships were observed, with the odds of cutaneous reactions increasing with the number of medications a patient is allergic to. Past dermatological disease was also found to have a significant association, as several studies and case reports have shown exacerbation of symptoms of dermatologic diseases such as dermatitis, psoriasis and dermatomyositis (38–42).

### Strength and Limitation

This study has some strengths. First, it included a large sample size because it was conducted at the largest tertiary hospital under the Ministry of Health (MOH), Malaysia; hence, due to the broad population of patients referred, the results can be applied to a broader population. The effect size estimates were not significantly altered when potential confounders were taken into account in the multivariable models, indicating that any residual confounding is unlikely to compromise the internal validity of the results.

There are limitations to this study. First, reporting of adverse events following immunisation was voluntary, not compulsory, hence it may not be representative of the actual size of cutaneous adverse reactions. Selective reporting by reporters and patients may result in the reporting of more serious reactions. Hence, milder reactions like localised reactions, which were more commonly reported elsewhere, were not captured in this study. Second, because healthcare providers collected the data at one point in time, this registry study comprises an incomplete record of follow-up for patients. Hence, it is unknown if patients with such cutaneous reactions have recovered with intervention or if they have been self-limiting, nor can we establish recurrences with successive vaccinations. Future research may attempt to set a follow-up duration and to get in touch with patients by email or phone calls after they receive a second dosage to address this issue. The morphological description of vaccine reactions is provider-dependent, which included non-dermatologists at Hospital Kuala Lumpur. The reactions were identified across various departments—emergency, inpatient and outpatient, as well as at vaccination centres by doctors and pharmacists. This diverse array of identification settings and personnel naturally introduces a level of variability in the morphological descriptions of the reactions. To enhance the reliability and depth of future studies, we recommend a more standardised approach to the identification and recording of cutaneous reactions post-vaccination. Incorporating dermatologists in the data collection process or providing specialised training to other healthcare providers involved in the identification of skin reactions could mitigate this limitation.

Cutaneous symptoms like urticaria, angioedema and/or maculopapular rash that were observed in this study could have been brought on by the host immune system or a reaction to nonsteroidal anti-inflammatory drugs, which are frequently used to treat pain and fever after vaccination, rather than by the vaccine itself. Moreover, this study was not able to capture which cases had allergist-confirmed allergic reactions, which could have contributed to a more meaningful outcome of the study.

Through a registry-based analysis without a denominator, we were unable to calculate the prevalence of cutaneous reactions after COVID-19 immunisation. Further population-level data will be needed to determine whether this is a true difference or related to reporting bias in order to overcome the possibility of confirmation bias, as providers were more likely to enter cases with severe or unusual presentations. Whether there is a true difference between men and women in the chance of developing a cutaneous reaction, or whether it may be the result of reporting bias or the fact that women make up the majority of the healthcare workforce remains to be determined.

## Conclusion

This case-control study found that a self-reported history of allergy to vaccines, food or medication was linked to an elevated likelihood of a self-reported cutaneous reaction. The majority of these cutaneous reactions were not serious and did not prevent the completion of the second dose of COVID-19 vaccination. Although more cutaneous skin reactions were observed after the first dose, frequently characterised as generalised pruritus and urticaria, symptoms were easily controlled with oral antihistamines and low doses of corticosteroids, which resolved manifestations in the majority of cases without the need for hospitalisation. Therefore, skin reactions do not present a barrier to completing the immunisation cycle, with the exception of acute hypersensitivity reactions (3, 43). Providing accurate immunisation advice requires an understanding of the range of cutaneous reactions to COVID-19 vaccines. Dermatologists and other healthcare professionals should be well-informed about the landscape of the most recent cutaneous reactions as data on vaccination reactogenicity continue to emerge, in order to address patients’ concerns.

## Figures and Tables

**Table 1:** Demographic, characteristics, clinical presentation and dermatologic findings of reported cutaneous adverse reaction following immunisation (*n* = 212)

	Comirnaty^®^ (*n* = 132)	Coronavac^®^ (*n* = 42)	Astrazeneca (*n* = 38)	Total (*N* = 212)
**Demographic characteristics**				

Age (years old)				
≤ 55	114 (67.1)	30 (17.7)	26(15.3)	170
> 55	18 (42.9)	12(28.6)	12 (28.6)	42
Age (median)	36 (30.0–44.5)	44.5 (29.0–59.0)	39 (29.0–63.0)	37 (29.0–49.5)
Gender				
Male	34 (55.7)	13 (21.3)	14 (23.0)	61
Female	98 (64.9)	29 (19.2)	24 (15.9)	151
History of heterologous COVID-19 vaccination				
No	125 (64.1)	38(19.5)	32 (16.4)	195
Yes	7 (41.2)	4 (23.5)	6 (35.3)	17
Prior SARS-COV-2 infection				
No	131 (62.7)	41 (19.6)	37 (17.7)	209
Yes	1 (33.3)	1 (33.3)	1 (33.3)	3
History of medication allergy				
No	104 (62.7)	33 (19.8)	29 (27.5)	166
Yes	28 (60.8)	9 (19.6)	9 (19.6)	66
History of vaccine allergy				
No	128 (63.7)	37(18.4)	36 (18.0)	201
Yes	4 (36.4)	5 (45.4)	2 (18.2)	11
History of any food allergy				
No	112 (60.6)	39 (21.0)	34 (18.4)	185
Yes	20 (74.1)	3 (11.1)	4 (14.8)	27
History of seafood allergy				
No	114 (60.3)	39 (20.6)	36 (19.1)	189
Yes	18 (78.3)	3 (13.0)	2 (8.7)	23
History of bronchial asthma				
No	116 (61.1)	39 (20.5)	35 (18.4)	190
Yes	16 (72.7)	3 (13.6)	3 (13.6)	22
History of sinusitis or rhinitis				
No	127 (100.0)	42 (100)	38 (100)	207
Yes	5 (100.0)	0	0	5
Comorbidities				
Epilepsy	1 (100.0)	0	0	1
Hypertension	6 (50.0)	3 (25.0)	3 (25.0)	12
Diabetes mellitus	7 (53.8)	3 (23.1)	3 (23.1)	13
Dyslipidemia	5 (62.5)	1 (12.5)	2 (25.0)	8
Ischaemic heart Disease	1 (20.0)	4 (80.0)	0	5
Hyperthyroid	1 (100.0)	0	0	1
Cancer	1 (33.3)	2 (66.7)	0	3
CKD	1 (100.0)	0	0	1
CVA	0	0	1 (100.0)	1

**Clinical presentation and dermatological findings**				

Severity				
Mild	113 (66.9)	32 (18.9)	24 (14.2)	169
Moderate	15 (53.6)	6 (21.4)	7 (25.0)	28
Severe	4 (26.7)	4 (26.7)	7(46.6)	15
Location of reaction				
Generalised	86 (60.6)	32 (22.5)	24 (16.9)	142
Localised	46 (65.7)	10 (14.3)	14 (20.0)	70
Proceed to receive second dose (after experience cutaneous reaction during first dose)				
No	13 (46.4)	5 (17.9)	10 (35.7)	28
Yes	54 (64.3)	16 (19.0)	14 (16.7)	84
Seriousness category				
Not serious	116 (63.4)	37 (20.2)	30 (16.4)	183
Caused or has prolonged hospitalisation	13 (72.2)	3 (16.7)	2 (11.1)	18
Life threatening	2 (20.0)	2 (20.0)	6 (60.0)	10
Caused permanent disability	1 (100.0)	0	0	1
Anaphylaxis reaction				
No	131 (62.7)	42 (20.1)	36 (17.2)	209
Yes	1 (33.3)	0	2 ( 66.7)	3
Proceed to receive booster dose (after experience cutaneous reaction during second dose)				
No	18 (58.1)	11 (35.5)	2 (6.4)	31
Yes	32 (80.0)	7 (17.5)	1 (2.5)	40
Dermatological morphology				
Urticaria	51 (65.4)	11(14.1)	16 (20.5)	78
Macular papular	37 (67.3)	13 (23.6)	5 (9.1)	55
Purpura	1 (50.0)	1(50.0)	0	2
Zoster	1 (100.0)	0	0	1
Erythematous	8 (80.0)	0	2 (20.0)	10
Pruritus	77 (63.1)	26(21.3)	19(15.6)	122
Rash of undocumented morphology	7 (70.0)	2(20.0)	1(10.0)	10
Eye swelling	17(63.0)	2 (7.4)	8 (29.6)	27
Lips swelling	2(28.6)	3 (42.8)	2(28.6)	7
Facial swelling	7 (53.8)	1(7.7)	5 (38.5)	13
Throat swelling	0	1(100.0)	0	1
Accompanied systemic reactions				
Lymph node swelling	1 (33.3)	1 (33.3)	1 (33.3)	3
Fatigue	0	1 (50.0)	1 (50.0)	2
Myalgia	4 (80.0)	0	1 (20.0)	5
Dizziness	20 (86.9)	1(4.35)	2 (8.7)	23
Headache	13 (92.9)	0	1 (7.1)	14
Fever	15 (53.6)	2 (7.1)	11 (39.3)	28
Chills and rigors	102 (60.7)	38 (22.6)	28 (16.7)	168
Nausea and vomiting	21 (72.4)	3 (10.3)	5 (17.2)	29
Diarrhoea	2 (50.0)	1 (25.0)	1 (25.0)	4
Chest pain	6 (75.0)	0	2 (25.0)	8

**Table 2:** Univariable logistic regression of risk factors associated with cutaneous reaction following immunisation of COVID-19 vaccine (*N* = 814)

	No. cutaneous reaction (*n* = 602)	Cutaneous reaction	Crude regression coefficient (SE)	Crude odds ratio (95% CI)	*P*-value
Age (years old)					
≤ 55	493 (74.36%)	170 (25.64%)			
> 55	109 (72.19%)	42 (27.81%)	0.11 (0.20)	1.12 (0.75, 1.66)	0.583
Gender					
Male	219 (78.21%)	61 (21.79%)			
Female	383 (71.72%)	151 (28.28%)	0.35 (0.17)	1.42 (1.00, 1.98)	**0.046**
Types of vaccine					
Comirnaty	367 (73.55%)	132 (26.45%)			
Coronavac	107 (71.81%)	42 (28.19%)	0.09(0.21)	1.09(0.72, 1.64)	0.675
Astrazeneca	128 (77.11%)	38 (22.89%)	−0.19(0.21)	0.83(0.55, 1.25)	0.363
History of COVID-19 vaccination within past 6 months (Repeated dose)					
No	398 (78.04%)	112 (21.96%)			
Yes	204 (67.11%)	100 (32.89%)	0.55(0.16)	1.74(1.26, 2.40)	**0.001**
History of heterologous vaccination					
No	545 (73.65%)	195 (26.35%)			
Yes	57 (77.03%)	17 (22.97%)	−0.18 (0.29)	0.83 (0.47, 1.46)	0.528
Prior SARS-COV-2 infection					
No	584 (73.64%)	209 (26.36%)			
Yes	18 (85.71%)	3 (14.29%)	−0.76 (0.63)	0.46 (0.14, 1.60)	0.224
History of any food allergy					
No	571 (75.53%)	185 (24.47%)			
Yes	31 (53.45%)	27 (46.55%)	0.99(0.27)	2.69(1.56, 4.62)	< 0.001
History of seafood or shellfish allergy					
No	577 (75.33%)	189 (24.67%)			
Yes	25 (52.08%)	23 (47.92%)	1.03 (0.30)	2.81 (1.56, 5.06)	0.001
History of medication allergy					
No	543 (76.59%)	166 (23.41%)			
Yes	59 (56.19%)	46 (43.81%)	0.94 (0.21)	2.55 (1.67, 3.90)	< 0.001
Number of medication allergy					
0	543 (76.59%)	166 (23.41%)			
1	34 (56.67%)	26 (43.33%)	0.92 (0.27)	2.50 (1.46, 4.29)	0.001
≥ 2	25 (55.56%)	20 (44.44%)	0.96 (0.31)	2.62 (1.42, 4.83)	0.002
History of vaccine allergy					
No	596 (74.78%)	201 (25.22%)			
Yes	6 (35.29%)	11 (64.71%)	1.69 (0.51)	5.44 (1.98, 14.88)	0.001
History of antibiotic allergy					
No	570 (75.10%)	189 (24.90%)			
Yes	32 (58.18%)	23 (41.82%)	0.77 (0.29)	2.17 (1.23, 3.80)	0.007
History of NSAID allergy					
No	574 (74.93%)	192 (25.07%)			
Yes	28 (58.33%)	20 (41.67%)	0.76 (0.30)	2.14 (1.18, 3.88)	0.013
Past dermatologic history (e.g. psoriasis, dermatitis)					
No	596 (75.16%)	197 (24.84%)			
Yes	6 (28.57%)	15 (71.43%)	2.02 (0.49)	7.57 (2.89, 19.76)	< 0.001
Bronchial asthma					
No	550 (74.32%)	190 (25.68%)			
Yes	52 (70.27%)	22 (29.73%)	0.20 (0.27)	1.22 (0.32)	0.449
Sinusitis or rhinitis					
No	594 (74.16%)	207 (25.84%)			
Yes	8 (61.54%)	5 (38.46%)	0.58 (0.58)	1.80 (0.58, 5.54)	0.310
Epilepsy					
No	597 (73.89%)	211 (26.11)			
Yes	5 (83.33%)	1 (16.67%)	−0.57 (1.09)	0.57 (0.06, 4.87)	0.604
Hypertension					
No	544 (73.12%)	200 (26.88%)			
Yes	58 (82.86%)	12 (17.14%)	−0.57 (0.33)	0.56 (0.30, 1.07)	0.079
Diabetes mellitus					
No	545 (73.25%)	199 (26.75%)			
Yes	57 (81.43%)	13 (18.57%)	−0.47 (0.32)	0.62 (0.33, 1.17)	0.139
Dyslipidaemia					
No	575 (73.81%)	204 (26.19%)			
Yes	27 (77.14%)	8 (22.86%)	−0.18 (0.41)	0.84 (0.37, 1.87)	0.661
IHD					
No	579 (73.66%)	207 (26.34%)			
Yes	23 (82.14%)	5 (17.86%)	−0.50 (0.50)	0.61 (0.23, 1.62)	0.320
Hyperthyroid					
No	599 (73.95%)	211 (26.05%)			
Yes	3 (75.00%)	1 (25.00%)	−0.05 (1.16)	0.95 (0.10, 9.15)	0.962
Cancer					
No	595 (74.00%)	209 (26.00%)			
Yes	7 (70.00%)	3 (30.00%)	0.20 (0.70)	1.22 (0.31, 4.76)	0.775
Smoker					
No	593 (73.94%)	209 (26.06%)			
Yes	9 (75.00%)	3 (25.00%)	−0.06(0.67)	0.95 (0.25, 3.53)	0.934

Notes: SARS-COV-2 = severe acute respiratory syndrome coronavirus 2; NSAIDS = non-steroidal anti-inflamatory drugs; IHD = ischaemic heart disease

**Table 3:** Risk factors associated with cutaneous reaction following immunisation of COVID-19 vaccine

	Adjusted regression coefficient (SE)	Adjusted odds ratio (95% CI)	*P*-value
History of seafood or shellfish allergy			
No			
Yes	0.75 (0.32)	2.11 (1.12, 3.96)	0.020
History of medication allergy			
No			
Yes	0.75 (0.23)	2.12(1.36, 3.31)	0.001
History of vaccine allergy			
No			
Yes	1.40 (0.53)	4.07 (1.43, 11.54)	0.008
Past dermatologic history (e.g. psoriasis, dermatitis)			
No			
Yes	1.70 (0.51)	5.48 (2.03, 14.78)	0.001

Notes: Constant = 0.037; Forward LR Multiple Logistic models was applied; Multicollinearity and interaction term were checked and not found; Model is fit with Hosmer-Lemeshow test P = 0.575, classification table = 75.2%
